# 2D and 3D Interdigital Capacitors and Bias Tees Technologies on MnM Interposer for mmWave Applications †

**DOI:** 10.3390/mi17020274

**Published:** 2026-02-23

**Authors:** Gabriel Griep, Robert G. Bovadilla, Leonardo G. Gomes, Luís Q. Cartagena, Gustavo P. Rehder, Ariana L. C. Serrano

**Affiliations:** Microelectronics Department, University of São Paulo, São Paulo 05508-010, Brazil; rgavidiab@alumni.usp.br (R.G.B.); leonardo.gomes@st.com (L.G.G.); lcartagena@usp.br (L.Q.C.); gprehder@usp.br (G.P.R.); aserrano@usp.br (A.L.C.S.)

**Keywords:** interdigital capacitor (IDC), bias tees, millimeter wave circuits, MnM technology

## Abstract

This paper presents two capacitors fabricated using the metallic nanowire membrane (MnM) interposer technology operating at mmWaves. Standard 2D interdigital capacitors (IDCs) are designed to operate up to 70 GHz, which presents a straightforward and non-complex fabrication. In comparison, this work also proposes an improved device that is more compact and exhibits large capacitance density, as high-performance vias enable the realization of high-depth capacitors. The fabrication process of 3D devices presents advanced maturity and innovation as it takes advantage of the porous nature of the interposer material to overcome the device complexity, and is also described in detail. Both capacitor types are modeled by a numerical lumped-element model that also considers parasitics. The 3D capacitors were successfully fabricated and characterized up to 70 GHz, displaying capacitance values between 30 fF and 160 fF and self-resonant frequencies in good agreement with mmWave applications. The quality factor of these devices, measured at 40 GHz, lies between 16 and 4, and the superficial capacitance density is between 4 pF/mm^2^ and 8 pF/mm^2^, showing that these devices are indeed promising for mmWave applications. These devices present considerably larger capacitance density compared to 2D traditional capacitors fabricated on the high-performance substrate, highlighting the advantage of 3D fabrication using nanowire growth. In addition, thin-film resistances are simulated and fabricated, projecting their functions as an RF-choke in a bias tee configuration using Ti thin film sputtering deposition step that is also part of the capacitors fabrication.

## 1. Introduction

In the millimeter-wave (mmWave) range, various unlicensed frequency bands are being selected for very high-bandwidth point-to-point communication links. The main focus of development is not solely on the unlicensed 60 GHz band [1], as applications such as automotive radars and road transport can also be found working at 77 GHz [2] and 120 GHz [3], respectively. Even at higher frequencies than 140 GHz, applications such as RF imaging, security, or medical applications can be found. Since mmWave communication requires a line of sight and free-space attenuation is high, transmissions are limited to a short range of tens of centimeters if only a single antenna is used.

This kind of system requires baluns, diplexers, filters, matching networks, and distribution networks for high-performance antenna arrays. It is clear that wavelength-based passive circuits (baluns, diplexers, filters, couplers, and antennas) should be implemented off-chip for reasons of efficiency and cost. One alternative solution is the use of 3D hybrid integration, either by placing the passive circuits above the integrated circuit (IC) or by using an interposer that supports passive circuits. However, for mmWave systems based on hybrid integration technologies, the overall system cost must be significantly reduced while maintaining adequate RF performance, since packaging and interconnection processes often represent a major fraction of the total system cost [4,5]. In this context, the development of a new generation of interposers capable of producing miniaturized and high-performance transmission lines and vias using a simple and low-cost manufacturing process represents a significant advancement for future 3D hybrid mmWave circuits and systems.

For mmWave and RF circuits, passive lumped components, such as capacitors, are an important element, being used in impedance matching, DC decoupling, filtering, biasing networks, DC blocking, etc. [6,7]. Traditionally, capacitors are realized in parallel-plate structures, where two metallic plates are separated by a thin insulator, metal–insulator–metal (MIM) devices. These devices are present both in monolithic technologies, such as (Bi)CMOS [8] and III–V processes [9], and in non-monolithic processes, such as interposers [10,11] and hybrid microwave integrated circuit (MIC) technologies, traditionally implemented using discrete or thin-film passive components on dielectric substrates [12]. MIM capacitors either require large areas to realize a target capacitance using standard process layers or extra fabrication steps using intermediate metal layers and thinner, high-K dielectrics to increase capacitance density. Multi-layer processes can use vertically stacked parallel plate (VPP) to increase capacitance density [13]; thus, vertical metal walls or mesh could be fabricated using multiple metal layers connected by vias. An example of these structures in CMOS technologies is the metal–oxide–metal (MOM) capacitors that result in compact devices that retain high capacitance density with high-frequency operation [14]. Single-layer processes can also employ interdigital capacitors (IDC) to realize capacitors [15,16], but in comparison with the MIM device, they show lower capacitance densities.

In this letter, a compact, interposer-embedded, 3D IDC operating at mmWave fabricated in a metal-filled nanoporous alumina membrane (MnM) is proposed. The technology was first introduced in [17] for the realization of slow-wave transmission lines. The advantage of this technology is the low complexity and low cost of the realization of Through Substrate Vias (TSVs), ref. [18]. Not only the fabrication of transmission lines and vias but also the precise modeling of these structures [19] and several other basic mmWave devices, such as 3D inductors [20], transformers [21], and crossovers [22], have achieved cutting-edge results. The availability of these small and high-performance vias operating up to 110 GHz [23] enables the fabrication of compact, mmWave-capable capacitors that require no complex or labor-intensive intermediate fabrication steps, and without the need for high-definition processes, maintaining a high density.

One of the main applications of capacitors in the field is the realization of bias tees for active circuits such as signal amplifiers, oscillators, and mixers. While the ideal RF choke relies on high-Q inductors, their realization in general presents narrow bandwidths and a high footprint area. As an alternative, we propose the thin-film Ti resistances that work as RF chokes, especially for small current biasing and ultrawide bandwidth.

This paper is organized as follows. In Section 2, characteristics of the device are explained, and the lumped-element model used for simulation and device layout is presented. Then, in Section 3, fabrication, measurement, and characterization results of the IDCs are given. In Section 4, a discussion on the proposed capacitors is drawn, followed in Section 5 by the concept of fabrication of the thin-film resistance—measurements of the resistance are presented, and how it can be applied on bias tees with simulation. Finally, a conclusion is drawn in Section 6.

## 2. Capacitor Device, Model, and Design

### 2.1. Device

**Figure 1:**
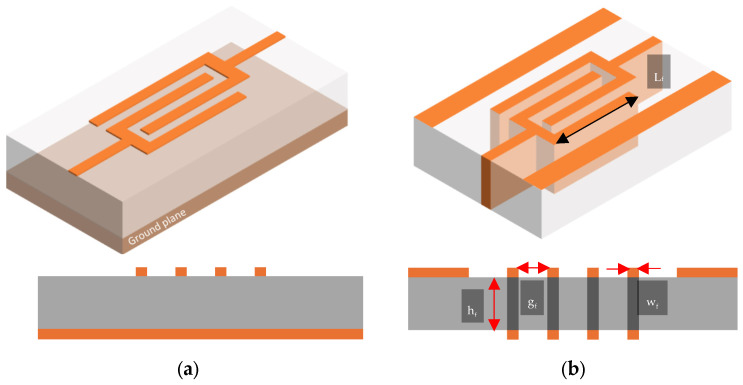
Layout of the test structure showing the capacitors (DUT). (**a**) The 2D microstrip line device and (**b**) the 3D CPW line.

The MnM interdigital capacitor proposed in this work is shown in Figure 1. Two-dimensional devices present only the top conductor surface thickness, which consists of a 3 μm Cu layer—they also present a microstrip with ground plane design (Figure 1a). In contrast to the typical fabrication, the 3D device presents thick walls that consist of through-substrate vias in the porous membrane of nanowires and are fabricated as CPW lines (Figure 1b). This way, the height (h_f_) of each wall is fixed at 50 µm, which corresponds to the height of the membrane. The other geometrical parameters are identical for both IDCs, and their capacitance can be controlled by changing the number of nanowire walls (n_w_), their length (L_f_), the width of the walls (w_f_), and the distance between the walls (g_f_).

For this structure, the capacitance of the device is directly proportional to the overlap area between the walls and the electrical permittivity and indirectly proportional to the distance between the walls. This way, for a 3D device, the structure can achieve higher capacitance density than a traditional interdigital capacitor.

To fabricate the walls, the nanopores of the alumina substrate were selectively filled with copper using an electrochemical process. Details about the fabrication process are shown in Section 3.

### 2.2. Model

**Figure 2:**
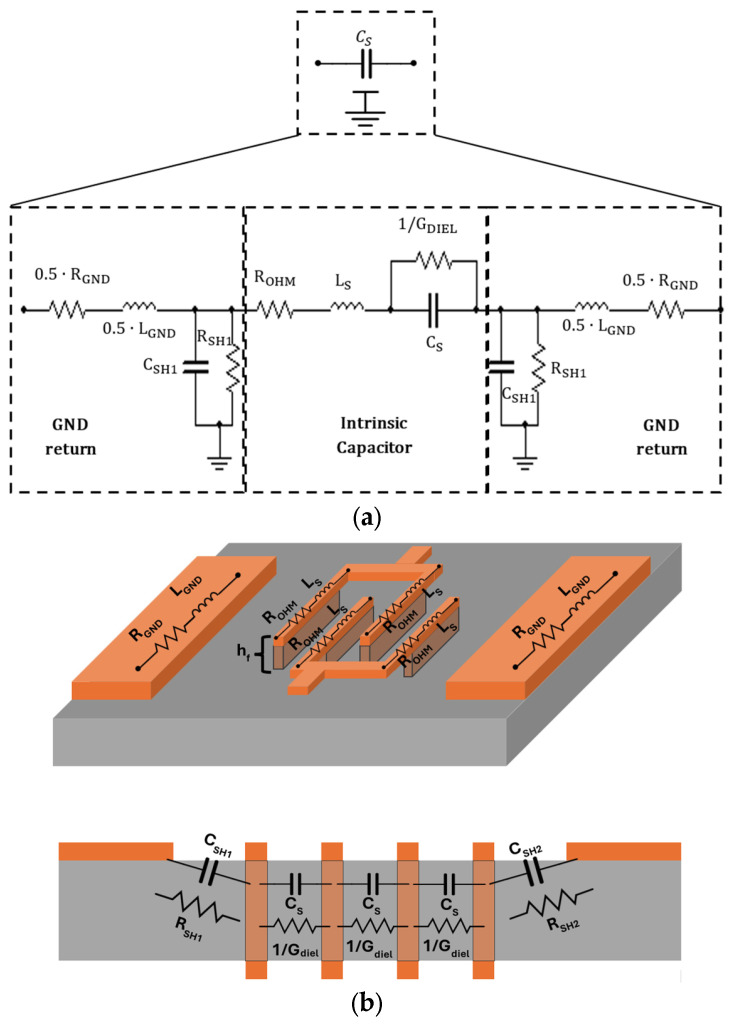
(**a**) MnM capacitor symbol and complete model, (**b**) 3D view and cross-section of the proposed interdigital MnM capacitor relating the layout to the proposed model.

The proposed lumped-element model and a view of the device are presented in Figure 2a and Figure 2b, respectively. The capacitor itself (C_s_), called an intrinsic capacitor, is modeled as a series RLC circuit, which encompasses the following: the series capacitance (C_M_), formed by the nanowire walls; the access inductance (L_M_); resistance (R_OHM_); and the dielectric resistance (1/G_diel_) in parallel with C_M_. Although the capacitor model is an RLC circuit, we need to evaluate the path return effects (GND return) for mmWave applications. For that reason, the model, shown in Figure 2a, represents the return path as a series-shunt RLCG network. It includes a series RL circuit modeling ground inductance and resistance (L_GND_ and R_GND_, respectively) and a shunt parallel RC circuit modeling the capacitive coupling and dielectric losses between the nanowire walls and the ground (C_SH_ and R_SH_, respectively). Consequently, the complete model can be described by a *π*-type equivalent topology including series parasitic elements and is represented using Y parameters. Equations (1)–(7) show the model variables and, where applicable, relate them to Y parameters or to the layout dimensions. This model is applied to both 2D and 3D devices, as both present the same conceptualization.

(1) $$C_{M}=\varepsilon_{0}\varepsilon_{r}\frac{h_{f}}{g_{f}}\cdot \left(n_{W}-1\right)\cdot L_{f}=\;\underset{f\to 0}{\lim}\frac{Im\{Y_{21}\}}{2\pi f}\;$$

(2) $$R_{tot}=\;R_{OHM}+\frac{1}{G_{diel}}+\;R_{GND}=-\frac{1}{Re\left\{Y_{21}\right\}}$$

(3) $$G_{diel}=\;\omega \cdot C_{M}\cdot tan\left(\delta \right)$$

(4) $$L_{M}=L_{SS}-M_{SGi}=\;\frac{1}{C_{M}\cdot {\left(2\pi SRF\right)}^{2}}$$

(5) $$L_{GND}=\;\frac{L_{GiGi}}{2}+\frac{M_{GiGi}}{2}-M_{SGi}$$

(6) $$C_{SHi}=\underset{f\to 0}{lim}\frac{Im\{Y_{ii}-Y_{ij}\}}{2\pi f}$$

(7) $$R_{SHi}=\;\frac{1}{{\omega \cdot C}_{SHi}\cdot tan\left(\delta \right)}=\frac{1}{Re\left\{Y_{ii}-Y_{ij}\right\}},\;i\in \{1,2\}$$

In Equation (1), C_M_ is the low-frequency capacitance, and ε_0_ and ε_r_ are the vacuum and substrate relative electric permittivity. In Equation (2), R_tot_ is the total series resistive component, and R_OHM_ and R_GND_ are the ohmic losses of the intrinsic capacitor and ground network, respectively. In Equations (2) and (3). G_DIEL_ is the equivalent dielectric loss of the substrate, and tan(δ) is the loss tangent for the substrate. In Equation (4), L_M_ is the total series inductance of the intrinsic capacitor, L_SS_ is the self-inductance of the intrinsic capacitor, M_SGi_ is the mutual inductance between the capacitor and ground, and SRF is the self-resonant frequency. In Equation (5), L_GND_ is the ground return inductance, L_GiGi_ is the self-inductance of one of the ground tracks, and M_GiGj_ is the mutual inductance between ground tracks. The equations for modeling the capacitive coupling and dielectric losses between the nanowire walls and the ground (C_SH_ and R_SH_, respectively) are shown in Equations (6) and (7). For the MnM substrate, ε_r_ = 6.7 and tan(δ) = 0.015.

The performance of a capacitor could be evaluated in function of the main capacitance (C_S_), its SRF, and quality factor (Q). Equations (8) and (9) give the Q and SRF as a function of model parameters.

(8) $$Q=\;\frac{Im\{Y_{12}\}}{Re\left\{Y_{12}\right\}}=\;\frac{\frac{1}{\omega C_{M}}-\omega L_{M}}{R_{tot}+\frac{1}{\omega C_{M}\cdot tan(\delta )}}$$

(9) $$SRF=\;\frac{1}{2\cdot \pi \cdot \sqrt{L_{M}C_{M}}}$$

## 3. Fabrication, Measurement, and Characterization

**Figure 3:**
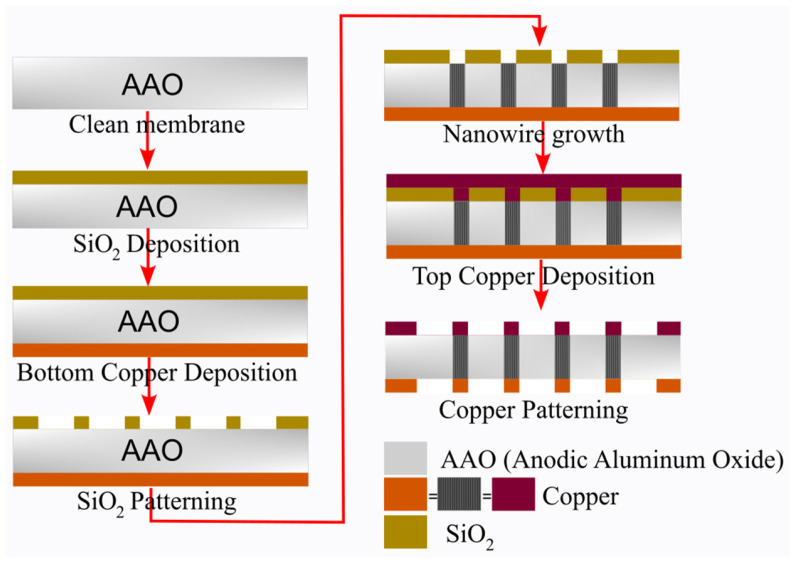
Fabrication steps of the proposed 3D interdigital capacitor on the MnM substrate.

**Figure 4:**
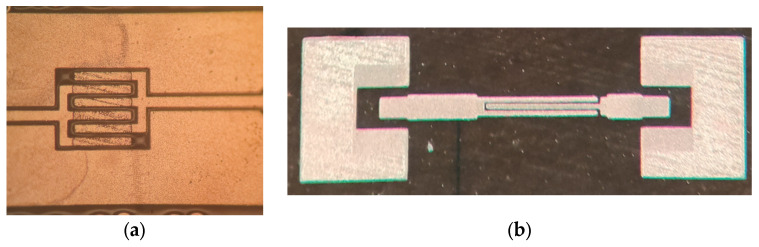
Fabricated interdigital capacitor devices on the MnM substrate: (**a**) 3D CPW capacitor and (**b**) 2D microstrip capacitor.

The fabrication process of the proposed three-dimensional (3D) interdigital capacitor on the MnM substrate is illustrated in Figure 3. The MnM substrate is based on a porous anodic aluminum oxide (AAO) membrane supplied by InRedox. The fabrication process begins with membrane cleaning using acetone and isopropanol, followed by the deposition of a 300 nm thick SiO_2_ mask layer by reactive magnetron sputtering. A copper seed layer is then sputtered onto the backside of the membrane. Subsequent steps include photolithographic patterning and buffered oxide etch (BOE) of the SiO_2_ layer, electrodeposition of nanowires, deposition and thickening of a copper layer on the top side of the membrane, and final patterning and etching of the copper layers. These steps define the top and bottom electrodes of the interdigital capacitor. In contrast to 3D CPW capacitors, microstrip 2D capacitors do not include the bottom copper deposition and nanowire growth, and basically consist of the top copper deposition. The final result of fabrication is presented in Figure 4.

For 2D devices, the sets of designs are shown in Table 1 with three different sets. Four different sets of 3D devices are also shown in Table 2. These were fabricated successfully to test both the extraction of model parameters and to validate the model itself. For 2D capacitors, the number of fingers was limited to three, since EM simulations presented parasitic resonance frequencies besides the SRF up to 70 GHz with similar lengths. As length becomes large enough, capacitive and inductive coupling introduce spurious spikes in S-parameters, as shown in [24].

The MnM capacitors were characterized up to 70 GHz using GSG probes (MPI Titan probes with 100 μm pitch) in a Cascade manual probe station EPS-150MMW (Cascade Microtech, Beaverton, OR, USA) and a Keysight PNA 5227B vector network analyzer (Keysight, Santa Rosa, CA, USA). A Line–Reflect–Reflect–Match (LRRM) calibration was conducted. To avoid short-circuiting the bottom of the transformers with the metallic chuck of the probe station, a 5 mm thick ROHACELL 51 IG foam was employed as a spacer.

The devices were simulated in Advanced Design System (ADS) Momentum and in the Ansys Q3D parasitic extractor to obtain the extracted values of inductances, resistances, and capacitances for the devices. The raw S-parameter data of the DUTs plus accesses were de-embedded in the ADS schematic to enable the extraction of the model parameters.

**Figure 5:**
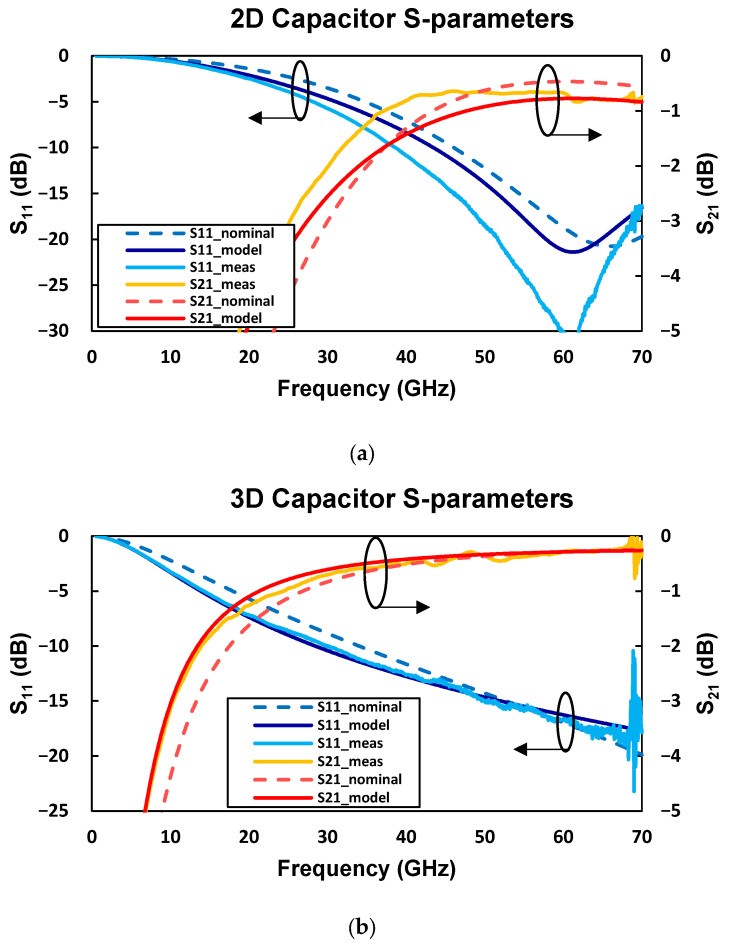
De-embedded S-parameters of the 2D and 3D capacitors, showing nominal (simulation), model (with the extracted parameters), and measured results. (**a**) A 2D capacitor with three fingers, $L_{f}=625\;\mu m$. (**b**) A 3D capacitor with four nanowire walls, $L_{f}=125\;\mu m$.

The de-embedded S-parameters for both types of capacitors are presented in Figure 5 together with the corresponding nominal (simulation), model (with the extracted parameters), and measurement results. The 2D capacitor corresponds to w_f_ = g_f_ = 10 µm and L_f_ = 625 µm, while the 3D capacitor corresponds to n_w_ = 4 and L_f_ = 125 µm. The results show good overall agreement between the model and the measurements across most of the analyzed frequency range, confirming the validity of the modeling approach and the parameter-extraction method. Some deviations are observed at the highest frequencies, mainly due to measurement-related limitations. In particular, the oscillation observed near 70 GHz is attributed to calibration uncertainties and measurement limitations as the frequency approaches the upper operating limit of the measurement equipment and is therefore considered a measurement artifact rather than an intrinsic device behavior.

The results comparing simulation and extraction from measurements in Table 3 show that the extracted capacitance is larger than expected according to simulations, and at the same time, the extracted results show higher losses. The authors attribute these divergences to process variations and to the de-embedding method used, specifically noting that the pad structures were not de-embedded, which adds parasitic effects and increases the apparent losses and capacitance in the measurements. It is highlighted that the five times shorter in length 3D capacitor presents three times higher capacitance and three times lower inductance—this occurs due to a higher capacitance area, expanded by the 3D walls that are fabricated through the substrate. The lower inductance is due to the shorter conductor length.

## 4. Discussion on IDCs

**Figure 6:**
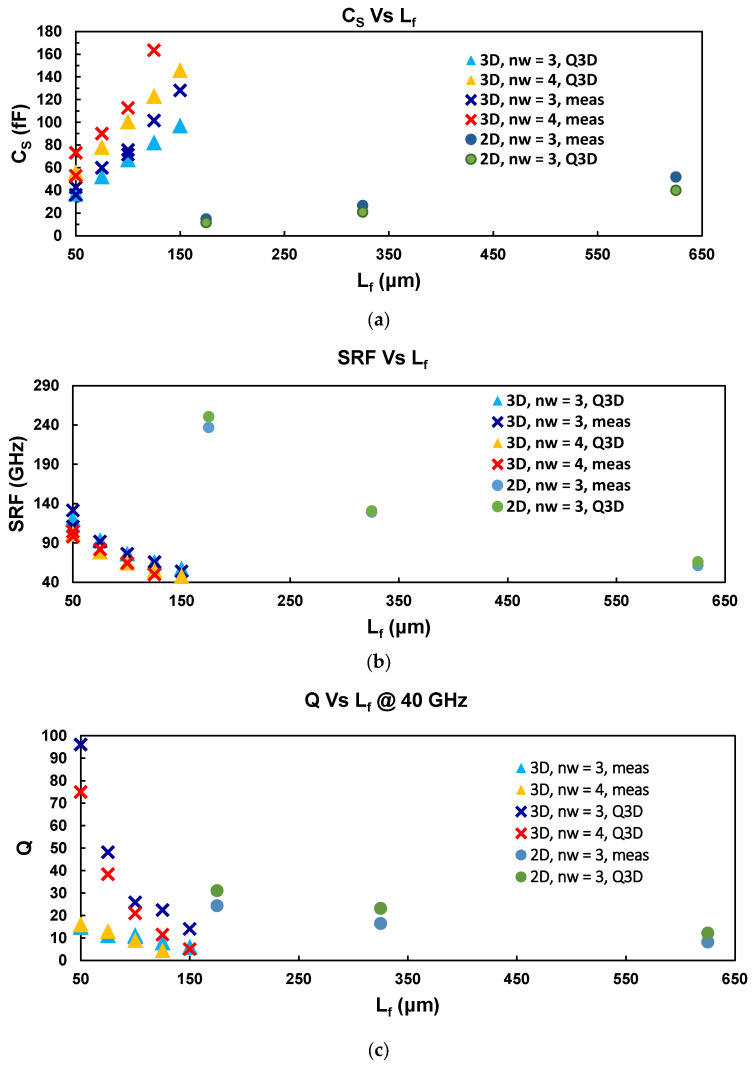
Comparison of three main performance parameters as a function of L_f_ and number of fingers: (**a**) intrinsic capacitance C_s_, (**b**) SRF, and (**c**) quality factor.

Results of C_S_, SRF, and Q for capacitors at 40 GHz with different values of L_f_ and n_w_ are shown in Figure 6. The results in Figure 6a show that the 3D capacitors with n_w_ = 3 exhibit values of Cs between 30 fF and 130 fF for L_f_ values ranging from 50 µm to 150 µm. On the other hand, the capacitors with n_w_ = 4 show Cs values ranging from 70 fF to 160 fF for L_f_ values between 50 µm and 125 µm. It is also highlighted that the much larger intrinsic capacitance of 3D devices, when compared to traditional 2D, as 2D 625 µm long IDCs are comparable with 3D 50 µm long in C_s_.

These results show that larger values of capacitance are obtainable by increasing n_w_ and L_f_. The correlation between extracted and simulated capacitance values is generally good, although deviations are observed for some devices. The agreement regarding the SRF (Figure 6b) remains very good for all device lengths. However, for Q (Figure 6c), the extracted values for devices with $L_{f}<100\;\mu m$ show a significant deviation from the simulated values.

The discrepancy in C_s_ may be attributed to manufacturing variations, such as nanowire wall thickness and a reduction in the gap between walls caused by over-etching during the patterning of the nanowire structures. This leads to a smaller gap between the IDC fingers, which leads to a stronger electric field and larger capacitance. This effect was also observed during the fabrication of devices with 5 µm, which resulted in short circuits by joining fingers and walls.

In the case of the quality factor, discrepancies are mainly attributed to the small value of the resistive losses of these devices, which become comparable to the probe contact resistance and to possible resistive via contacts introduced during on-wafer measurements.

These parasitic contributions introduce an additional series resistance, which is approximately constant for all measurements, while the intrinsic device resistance increases with the finger length $L_{f}$. For short devices ($L_{f}<100\;\mu m$), device resistance is very small, and the total resistance is dominated by the series resistance, leading to an underestimation of the extracted Q values.

As $L_{f}$ increases, the intrinsic resistive losses become larger and the relative impact of the contact resistance decreases, improving the agreement between simulated and extracted values. For the much larger 2D reference devices, the intrinsic resistive losses are sufficiently high for this approximation to become accurate.

**Figure 7:**
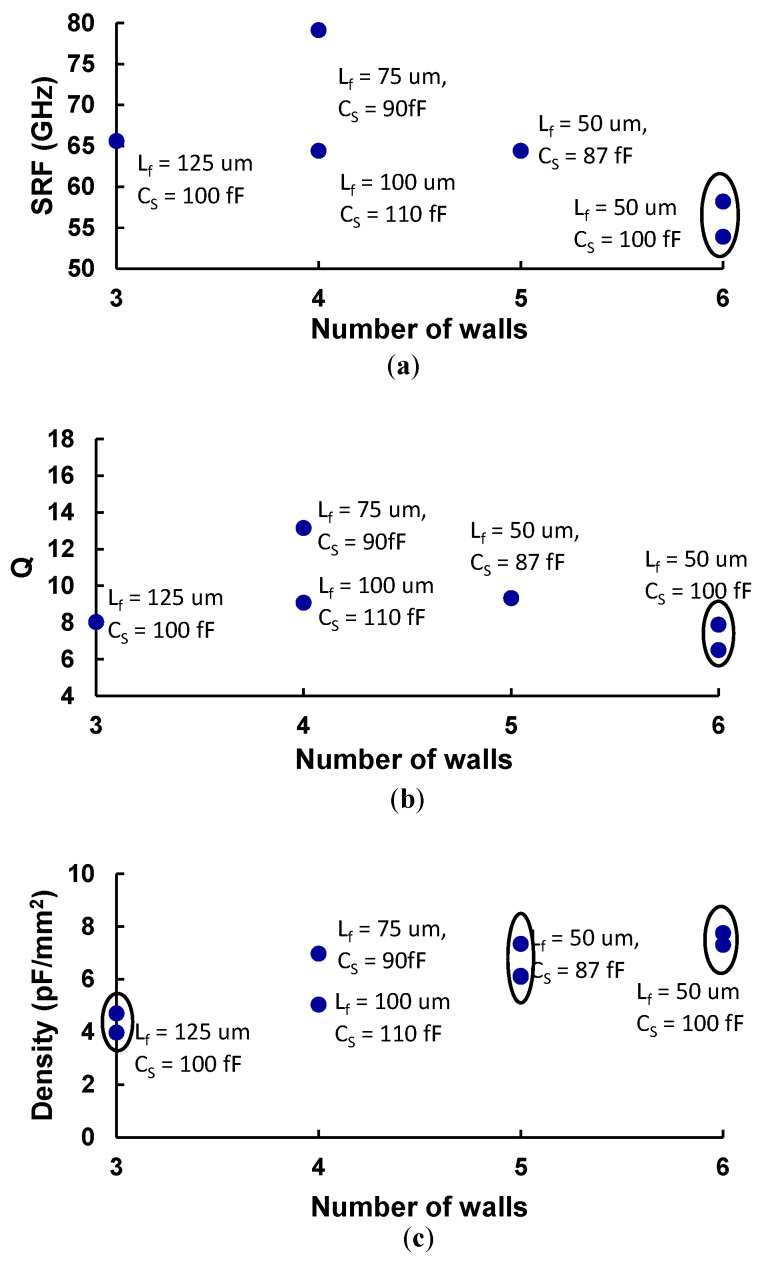
Comparison of three main performance parameters as a function of L_f_ and number of walls: (**a**) self-resonant frequency, (**b**) quality factor, and (**c**) capacitance density.

Considering C_s_ values close to 100 fF, Figure 7 shows the extracted values for SRF (Figure 7a), Q at 40 GHz (Figure 7b), and capacitance density (Figure 7c) for different values of n_w_ and L_f_, only focusing on 3D devices. These devices exhibit a peak Q of 11.58 and an SRF of 79 GHz, demonstrating their suitability for mmWave applications. It can also be observed that the capacitors show better performance for shorter L_f_, which may be attributed to the fact that longer lengths introduce more parasitic effects.

The results for the surface capacitance density for capacitors of L_f_ = 50 µm for n_w_ between 3 and 6 show values between 4 pF/mm^2^ and 8 pF/mm^2^. Table 4 contains the capacitance density of MIM, MOM, planar IDC, and MnM IDC.

The MnM IDC has about 10 times the capacitance density (surface) when compared to standard IDCs on traditional Si substrates, 2.5 to 5 times compared to 2D IDCs on the MnM substrate, and 6 to 3 times the surface density of interlayer MIM devices on interposers. However, compared to thin-film MIM devices, the MnM 3D IDC shows about 30 times less capacitance density. The capacitance density of monolithic capacitors is also included for reference, where it can be seen that these devices display about 1000 times denser than devices on interposers or MICs. Thus, the MnM 3D IDC is more compact than devices on comparable technologies while operating at mmWaves.

## 5. Application Study Case: Thin-Film Resistances and Bias Tee Application

One of the main applications of capacitive elements in the mmWave field is the realization of bias tees. Although commercial bias tees are widely available for mmWave instrumentation, designs for integrated mmWave systems are more challenging and often present narrow bands using spiral inductors and λ/4-long transmission lines as RF chokes. Therefore, it is proposed to fabricate and characterize an alternative by using high-resistance thin-film lines that work as RF chokes and can be integrated along the capacitors that work as DC blocks. Before the fabrication process of Cu deposition mentioned in Section 3, a thin Ti layer is deposited by sputtering for 50 s, corresponding to around 1.5 nm thickness, for the primary function of adherence between the MnM substrate and Cu conductor. Taking advantage of this thin-film, the proposed resistances use this Ti layer as the main conductor.

**Figure 8:**
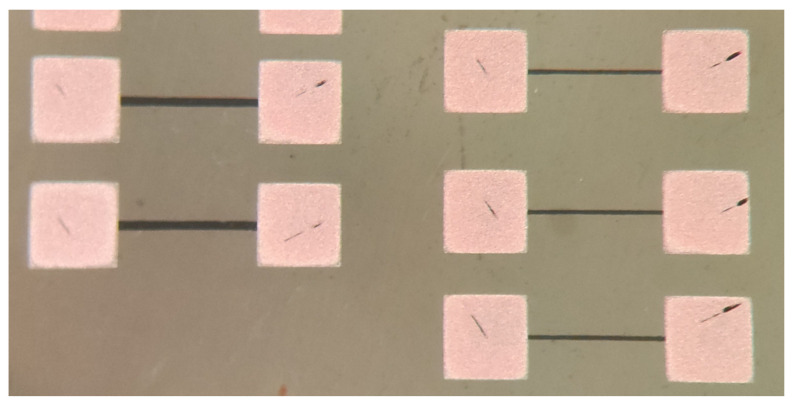
Fabricated and measured thin-film Ti resistances.

The fabricated devices are shown in Table 5. The manufactured devices present a fixed length of 285 μm. Lines fabricated as shown in Figure 8 were measured with a bench multimeter and DC probes in a Cascade manual probe station (EPS-150MMW).

Due to the small thickness of the thin-film (1.5 nm), very limited current is supported. Indeed, current densities on the order of 10^6^ A/cm^2^ represent a threshold for failure caused by electromigration and subsequent short-circuiting [25]. Using a conservative limit of 0.5 × 10^6^ A/cm^2^ and a width of 30 μm, the maximum current supported for biasing results in 225 μA.

**Figure 9:**
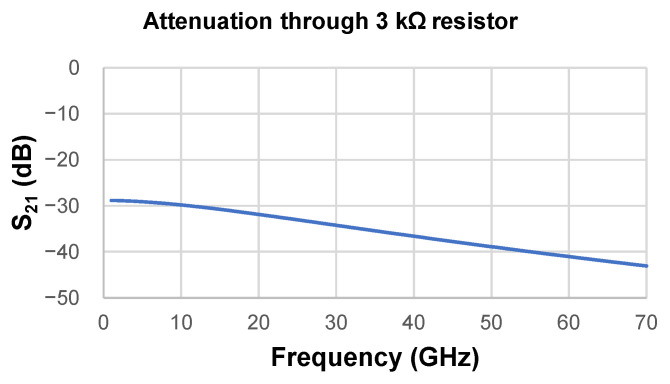
Attenuation of the 3 kΩ resistor simulated on momentum.

The resistance value of 3.0 kΩ resulted in an attenuation between 28 and 42 dB up to 70 GHz, stating its isolation function in wideband computed by Momentum Simulation on ADS, as shown in Figure 9, providing enough isolation between RF signals and DC sources.

**Figure 10:**
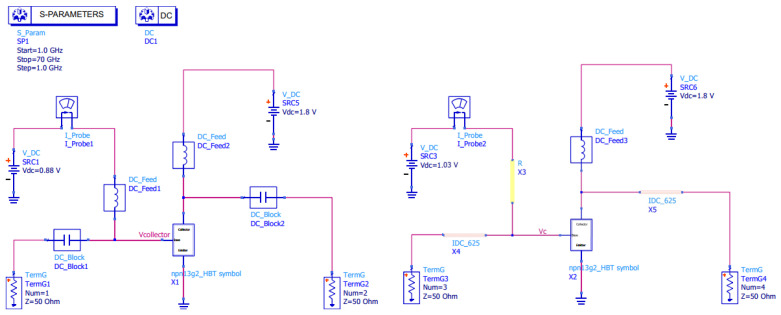
Bias tee compared with ideal components (**left**) and capacitor and RF choke proposed by this paper (**right**).

**Figure 11:**
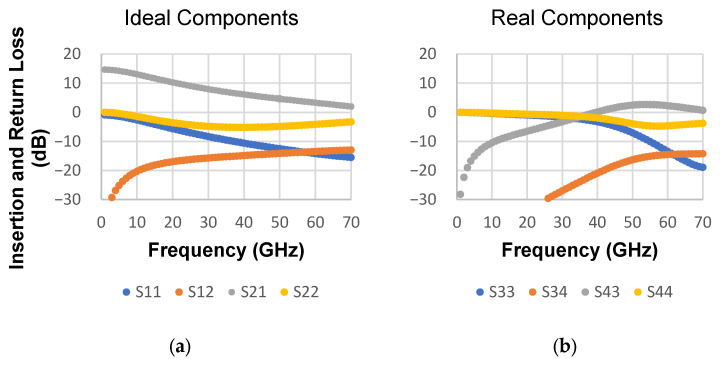
S-parameters for (**a**) a transistor biased with an ideal bias tee and (**b**) with 2D IDC and thin-film resistor in the base.

To highlight an application of this bias tee in a feasible context, the open-source HBT model NPN13G2 provided by IHP [26] is used. With the SPICE model on ADS schematic simulation, a scenario in a CE configuration with an ideal bias tee is compared with one bias composed of (1) 30 μm wide and 285 μm long resistance and (2) L_f_ 625 μm, w_f_ 10 μm, g_f_ 10 μm, n_w_ 3 walls, and a 2D capacitor—both simulated on Momentum—as shown in Figure 10. The base current is 51.4 μA, which is below the reference value of 225 μA. In the real-world scenario, the thin-film resistor can be used in an HBT base, where current remains low enough, and by the collector, an ideal choice is an inductor to handle mA-order currents. In terms of HBT performance regarding S-parameters, this is exhibited in Figure 11, comparing both scenarios. As expected, the real-world scenario biasing only works beyond 50 GHz, since it is the range where the capacitance is large enough to act as a DC block. The maximum gain for the proposed real-world scenario occurs at 54 GHz, with S_43_ equal to 2.67 dB, whereas the ideal scenario presents S_21_ of 4.09 dB. The loss is mainly attributed to the IDC.

**Figure 12:**
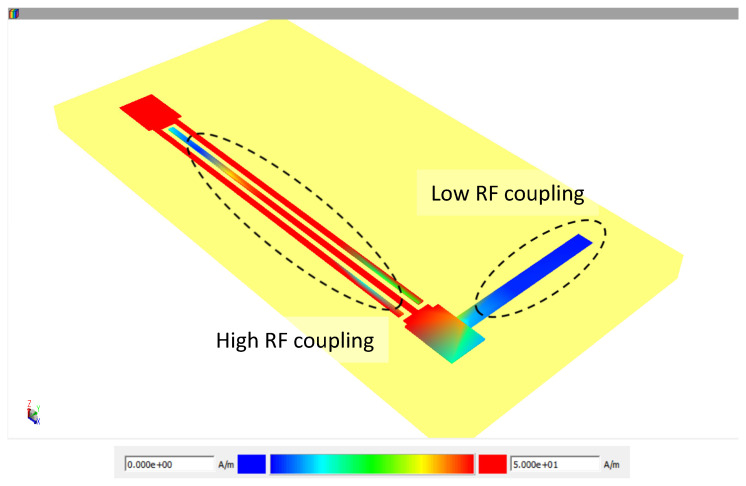
Electromagnetic field at 40 GHz over the components of the proposed bias tee with high coupling between IDC fingers and isolation through the thin-film resistor simulated on ADS.

Figure 12 exhibits a simulation with both components integrated in the layout tool and simulated in momentum on ADS—it is possible to observe a high transmission between fingers of both ends of the capacitor and a high isolation over the thin-film resistor. While simulation presents a functional bias tee, the fabrication of the entire structure is currently in progress in the mmWave Center at USP.

## 6. Conclusions

In this paper, a low-cost technology is used to fabricate a novel, 3D, substrate-embedded IDC that features a quality factor and self-resonant frequency compatible with mmWave applications, as well as higher capacitance density than other non-monolithic capacitors. This structure is, to the author’s knowledge, the first occurrence of a 3D interdigital capacitor that is not fabricated on monolithic technologies that can operate in mmWaves with adequate performance. These devices, thus, can bridge the gap between integrated, mmWave-oriented capacitors and interposer-based, DC filtering-oriented capacitors. In order to compare to traditional devices in the MnM material, which is promising in these frequencies, those were compared to 2D devices, and the novel material proved to increase capacitance and quality factor significantly. As these devices in such lengths and capacitances can be used as bias tees, it is also proposed that resistances be fabricated at the same step to introduce RF chokes in a straightforward way, which are expected to be suitable for small current biasing while providing isolation of around 30 dB. Although capacitors and resistors were fabricated as separate structures, a case study shows by simulation that this approach can be used for HBT base biasing. Fabrication with the integration of the entire structure is in progress in the mmWave Center at USP for future work.

## Figures and Tables

**Table 1:** Set of 2D-manufactured devices.

Set	n_w_	L_f_ (μm)	g_f_ (μm)	w_f_ (μm)
1	3	175	10	10
2	3	325	10	10
3	3	625	10	10

**Table 2:** Set of 3D-manufactured devices.

Set	n_w_	L_f_ (μm)	g_f_ (μm)	w_f_ (μm)
1	2	50, 150, 250, and 350	10	10
2	3	50, 150, 250, and 350	10	10
3	5	150	10, 15, and 20	5, 10, 15, and 20
4	6	150	10, 15, and 20	5, 10, 15, and 20

**Table 3:** Comparison between nominal and extracted parameters.

		C_S_ (fF)	C_SH1_ (fF)	C_SH2_ (fF)	L_S_ (pH)	SFR (GHz)	R_TOT_ (Ω)
2D: L_f_ 625 μm, w_f_ 10 μm, g_f_ 10 μm, n_w_ 3 walls	Nominal	39.9	60.1	23.1	149.8	65.10	2.12
	Extraction	51.8	99.4	45.5	130.5	61.20	0.30
3D: L_f_ 125 μm, w_f_ 10 μm, g_f_ 10 μm, n_w_ 4 walls	Nominal	123.25	9.73	9.88	47.9	55.05	1.065
	Extraction	163.6	14	12	47.9	49.80	1

**Table 4:** Comparison of mmWave capacitors in the literature and this work in nF/mm^2^.

MIM (CMOS [3,8], MMIC [4])	MOM (CMOS [8])	MIM (Interposer)	IDC	MnM 2D IDC (This Work)	MnM 3D IDC (This Work)
1–25 (“free” device) 21 (high K)	2.5 (130nm) 9 (14nm)	0.0015 [11] (free) 0.2 [7]-0,4 [4] (thin film)	5.8 × 10^−4^ [10] 6.4 × 10^−4^ [11]	0.0016–0.0017	0.004–0.008

**Table 5:** Set of thin-film resistances.

Sample	20 μm	30 μm
1	3.9 kΩ	2.8 kΩ
2	4.2 kΩ	3.1 kΩ
3	4.5 kΩ	3.1 kΩ
4	5.0 kΩ	2.9 kΩ
Average	4.4 kΩ	3.0 kΩ

## Data Availability

The original contributions presented in this study are included in the article. Further inquiries can be directed to the corresponding authors.
